# An intra-articular ganglion cyst in a patient with juvenile idiopathic arthritis

**DOI:** 10.1186/1546-0096-12-14

**Published:** 2014-04-23

**Authors:** Donna Y Deng, Keolamau Yee, William Burkhalter, Kelley Chinen Okimoto, Kevin Kon, David K Kurahara

**Affiliations:** 1 Department of Pediatrics, John A. Burns School of Medicine, University of Hawaii, Honolulu, Hawaii

**Keywords:** Intra-articular ganglion cyst, Juvenile idiopathic arthritis

## Abstract

We report an intra-articular ganglion cyst (IAGC) presenting as knee pain and a mass in a patient with longstanding Juvenile Idiopathic Arthritis (JIA). We could not find a similar case of an IAGC occurring in the knee of JIA patients in the literature. IAGC may need to be included as a possibility in patients with inflammatory arthritis with new-onset knee pain, especially in those with a palpable mass. MRI was useful in distinguishing IAGC from more worrisome causes of a knee mass. Orthopedic input was helpful in diagnosis and treatment. In addition, methotrexate therapy was effective in bringing about a long-lasting remission.

## Background

Juvenile Idiopathic Arthritis (JIA) is a heterogenous disorder characterized by chronic inflammatory arthritis and exacerbations that present with joint pain, swelling, and morning stiffness, but should not present with a knee mass [1,2]. Besides an acute flare of the disease causing joint discomfort, other causes of pain in JIA patients may include infection, trauma, tumor, or associated orthopedic conditions [2,3]. We present the first patient, to our knowledge, with JIA who developed a knee mass due to an intra-articular ganglion cyst (IAGC). IAGC has been reported as a cause of knee pain in adults without arthritis [4,5], but in children without arthritis, only a few cases have been reported [6-8].

When this patient developed a knee mass, it was very concerning to the family and care providers because of the extensive family history of cancer and the previous years of treatment with methotrexate. We could not find a similar case report of IAGC in JIA patients and this possibility should be considered in patients who present with a knee mass, particularly if their arthritis is flaring up. This knee mass and pain was responsive to needle aspiration and methotrexate therapy.

## Case presentation

A 14-year-old Caucasian girl with JIA diagnosed at 2 years of age returned to our pediatric rheumatology clinic with complaints of increased morning stiffness of both knees and a mass of the left knee. Three weeks prior to the onset of knee stiffness, she noticed a mass on the lateral side of her left knee. She had decreased knee flexion and pain with walking that was greater on her left side. She reported a painful snap when she moved her left knee from a flexed to extended position, but no instability or locking. There was no warmth, erythema, interval growth, or fluctuance of the mass. She also did not have any systemic symptoms including fever, chills, or recent weight loss.

Her past medical history was significant for extended oligoarticular JIA with 5 joint involvement and a positive Anti-nuclear antibody (ANA at 1:80), HLA-B27 negative, and rheumatoid factor (RF) negative. Over the first two years of her illness, she required only NSAIDs until she developed a photosensitive rash with Naproxen. Over the next 6 years, she needed methotrexate and ibuprofen intermittently for flare ups and was maintained on these medications for 1 to 2 year intervals and then tapered off. She had been disease free for three years when she presented with this new knee mass. She had a brief episode of mild iritis earlier in the course of her disease with full resolution while on methotrexate. Our patient lived on a neighboring island far from pediatric rheumatology services. When we were informed about the patient’s knee mass, we were extremely concerned because of her strong family history of cancer and her earlier treatment with methotrexate. Her family had a range of cancers including liver, pancreatic, stomach, throat, lymphoma, melanoma, non-Hodgkin's lymphoma, and glioblastoma in different members of the family.

On examination, there was a 1.5 × 2.0 cm well-circumscribed, and non-tender mass. The mass was on the lateral superior aspect of the left patella/quadriceps tendon just above the kneecap and crossed over the top of the femur. As the knee was moved from flexion to extension, there was a palpable and painful snap when the muscles moved past this mass over the tendon. The knee had full range of motion, was stable to varus/valgus stress, and had no medial or lateral joint line tenderness. There was also swelling in both knees. McMurrays and Lachmans test were negative, and capillary refill was brisk in all digits.

**Figure 1:**
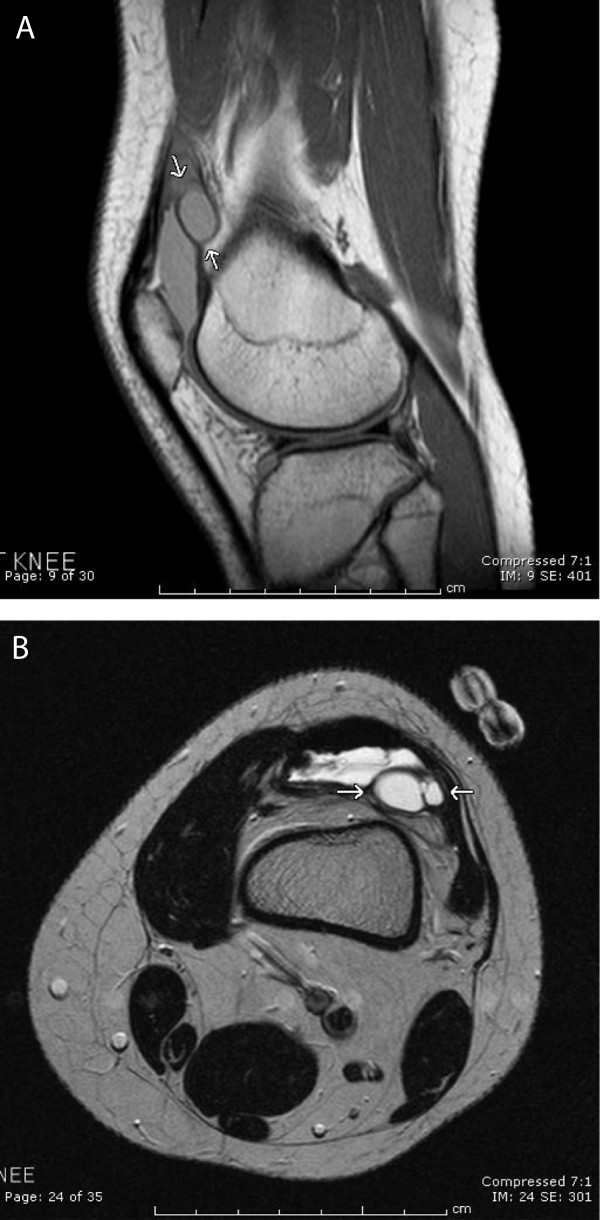
**MRI of intra-articular ganglion cyst. (A)** Axial T2-weighted image shows a mildly septated T2 hyperintense fluid intensity lesion within or projecting into the superolateral aspect of the knee. **(B)** Sagittal proton density weighted image shows a slightly elongated fluid intensity area adjacent to or within the upper aspect of the joint space.

Diagnostic imaging included a normal knee radiograph. Magnetic resonance imaging (MRI) of the left knee showed a moderate joint effusion with an approximately 1.5 cm diameter loculation slightly offset laterally which corresponded to the patient’s knee mass (Figure 1). Orthopedics diagnosed an IAGC and aspirated 0.5 ml of off-white, translucent thick material from the left knee mass. Cytology of the fluid revealed lymphocytes and macrophages without malignant cells. The combination of methotrexate and the IAGC aspiration resolved the patient’s knee pain, swelling, stiffness, and snapping symptoms she had experienced with the IAGC.

## Discussion

Knee pain and swelling is a common complaint in patients with JIA, but other causes besides an acute exacerbation of arthritis should be evaluated. In our case, the patient had a worrisome history that includes a long standing history of JIA, a strong family history of cancer, and a prior course of methotrexate therapy that prompted further investigation of her new knee mass. Our patient’s extensive family history of cancer was quite concerning since several studies reported an increased baseline risk of malignancy, including hematologic and solid tumors, in patients with JIA [9-11]. A search of the literature has suggested an increased incidence of malignancy in JIA related to methotrexate therapy [10-12]. The patients diagnosed with malignancy after methotrexate therapy differ from our patient because they had systemic or polyarticular JIA and likely needed longer and higher doses of methotrexate to manage a greater severity of disease [1,3]. Some of these patients may also have received an additional biologic agent, including TNF alpha inhibitors, which may cause added risk for malignancy. Our patient has done well with methotrexate, and has not required therapy with biologics. However, it was due to these alarming factors in our patient’s history that led to prompt referral to a Pediatric Orthopedist and the unique finding of an IAGC as the cause of our patient’s knee pain and swelling.

IAGC is a benign tumor-like lesion surrounded by a dense connective tissue capsule filled with gelatinous fluid rich in hyaluronic acid and other mucopolysaccharides [13,14]. The prevalence of IAGC is approximately 0.1-1.3% in the literature [4,5,13], which is based on adult studies using MRI and arthroscopy, and more common in males. There have only been a few case reports of children without arthritis with IAGC who presented with knee pain [6-8]. Possible causes of IAGC include synovial herniation through a defect in the joint capsule or tendon sheath, mucinous degeneration of connective tissue from repeated minor trauma, or proliferation of pluripotent mesenchymal cells [13-15]. IAGC are most commonly associated with the anterior cruciate ligament (ACL), but can also occur at the posterior cruciate ligament (PCL), infrapatellar fat pad, posterior joint capsule, from chondral fractures, or with subchondral bone cysts [5,13,14,16]. Clinical symptoms are nonspecific and depend on cyst size and location. These include knee pain, clicking or popping sensations, a palpable mass, and decreased range of motion [14,16]. Many patients with IAGC had lesions discovered on MRI or at arthroscopy after being evaluated for these non-specific symptoms [5,15,16]. These overlapping symptoms may mimic similar common complaints in patients with JIA. IAGC appear as cystic masses on MRI, have a homogenous low signal intensity on T1-weighted images and high signal intensity on fluid-sensitive and T2-weighted images [13,14,17].

There has been one previous report describing a ganglion cyst in the ankle of a JIA patient with clinically active arthritis visualized with the aid of an ultrasound [18]. It is interesting to note that both these cases had active arthritis present when the ganglion was found. In our case, the patient presented with complaints of knee pain and the IAGC was identified on MRI. Current treatment of IAGC includes arthroscopic resection, or ultrasound- and CT-guided joint paracentesis [15,19]. It is still possible to have some IAGC recurrence after paracentesis, but recurrence is rare after resection. Our patient’s IAGC responded well to aspiration and the patient was re-started on methotrexate to manage her flare-up of JIA when malignancy was not identified. Due to the IAGC’s close proximity to the skin surface in the superior patellar pouch, it responded well to this therapeutic approach; however it is possible that deeper ganglions may need surgical resection.

## Conclusion

We present the first case to our knowledge of an IAGC in the knee of a patient with active JIA. Joint discomfort is a common complaint in JIA patients, but symptoms of knee clicking, popping, and snapping with the presence of a mass may indicate the need for further evaluation by our orthopedic colleagues. There may be need for an MRI to better define the lesion, and if the JIA is flaring, appropriate disease modifying medications may need to be started.

## Consent

This project was reviewed by our institutional research review office and felt to be a case report and therefore exempt from full committee review.

## Abbreviations

JIA: Juvenile idiopathic arthritis; IAGC: Intra-articular ganglion cyst; PCL: Posterior cruciate ligament; ANA: Anti-nuclear antibody; HLA-B27: Human leukocyte antigen-B27; MRI: Magnetic resonance imaging; RF: Rheumatoid factor.

## Competing interests

No external funding was secured for this study. The authors have no financial relationships relevant to this article or competing interests to disclose.

## Authors’ contributions

DD cared for the patient and wrote most of the manuscript. DK was the senior physician responsible for care of the patient, and also assisted in revising much of the manuscript. KK read the MRI and contributed the figures and his impressions. KC first evaluated the patient, documented most of the family history, and helped revise the manuscript. KY assisted with development of the case report and in editing the final report. WB was instrumental in diagnosis and treatment of the patient and reviewing the case report. All authors read and approved the final manuscript.

## Authors’ information

DD is a senior pediatric resident interested in Pediatric Rheumatology, DK is a practicing Pediatric Rheumatologist and Medical Director of Pediatric Rheumatology at the University of Hawai‘i, KK is a practicing Pediatric MRI radiologist at the Children’s Hospital in Honolulu, KC is a senior pediatric resident at our Children’s Hospital interested in Pediatric Hematology/Oncology, KY is a senior undergraduate student doing research in our institution, and WB is a Pediatric Orthopedist at our Children’s hospital.
